# Laboratory simulation of uranium metal corrosion in different soil moisture regimes

**DOI:** 10.1016/j.mex.2020.100789

**Published:** 2020-01-17

**Authors:** Qinku Zhang, Steven L. Larson, John H. Ballard, Pohlee Cheah, Xianchun Zhu, Heather M. Knotek-Smith, Fengxiang X. Han

**Affiliations:** a Department of Chemistry and Biochemistry, Jackson State University, 1400 J. R. Lynch Street, Jackson, MS 39217, USA; b School of Civil Engineering and Shaanxi Key Laboratory of Ecological Restoration in Shanbei Mining Area, YulinUniversity, Yulin, Shaanxi 719000, China; c U.S. Army Engineer Research and Development Center, 3909 Halls Ferry Rd., Vicksburg, MS 39180-6199, USA

**Keywords:** Laboratory simulation of uranium metal corrosion in different soil moisture regimes, Uranium metal, Uranium dioxide, Uranium trioxide, Corrosion, Soil

## Abstract

A novel laboratory simulation system has been developed for the study of the corrosion of uranium metal in soils. Corrosion and transportation of depleted uranium (DU) as the metal undergoes weathering as a buried material within the soil environment. The corrosion of uranium metal in soil was not well understood due to the gas-liquid-solid phase of the soil. This study presents a novel method to investigate the change of uranium species during the process of process of oxidation of metallic uranium in these environments. Compared with other techniques used for the study of environmental corrosion of metals in soils, this method has the advantage of low secondary uranium pollution, no energy consumption, and ease of operation. The simulation system has been used for the following studies:

• Simultaneously simulate the corrosion of uranium metal in different soil moisture regimes
• Study the influence of biogeochemical factors on the corrosion of uranium metal
• Investigate the change of uranium species during oxidation

## Graphical abstract

**Figure:**
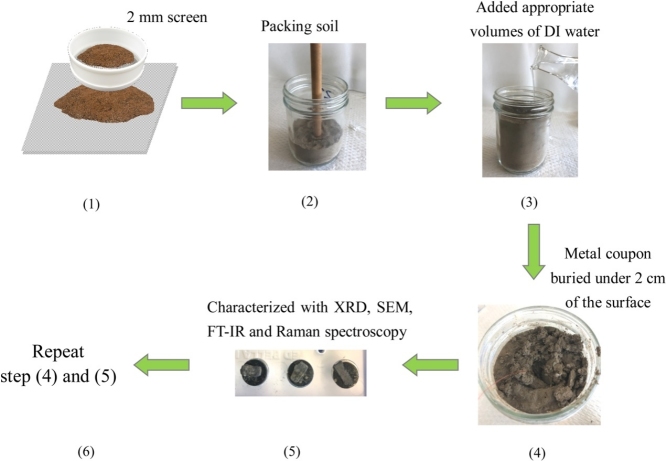

**Specification Table**

**Table:** 

Subject Area:	Environmental Science
More specific subject area:	Heavy metal pollution and control
Method name:	Laboratory Simulation of Uranium Metal Corrosion in Different Soil Moisture Regimes
Name and reference of original method:	S. Handley-Sidhu, P.J. Worsfold, F.R. Livens, D.J. Vaughan, R. Alvarez, M.J. Keith-Roach. Biogeochemical Controls on the Corrosion of Depleted Uranium Alloy in Subsurface Soils, Environmental Science & Technology, 43 (2009) 6177-6182. [2]
	F.X. Han, W.L. Kingery, J.E. Hargreaves, T.W. Walker, Effects of land uses on solid-phase distribution of micronutrients in selected vertisols of the Mississippi River Delta, Geoder, 142(2007) 96–103. [12]
Resource availability:	N/A

## Method details

### Overview

Production of depleted uranium (DU) metal is a means of reducing the uranium hexafluorophosphate waste produced during the process uranium enrichment. This metal has unique properties as a result of the high density and reactivity of metallic uranium. More than 17 countries are thought to have used these munitions as part of their military weapons systems [1]. In addition to military use, DU metal is used in a variety of civilian products. It is used as ballast in ships and airplanes. Boat keels, flywheels, gyroscopes and helicopter rotors are manufactured using depleted uranium. DU metal is and effective shielding material and is used as radiation shielding in radioactive material transport containers [2,3].

Kinetic energy penetrator weapons have been used in Iraq, Bosnia, Herzegovina, Kosovo/Serbia [4,5] and at military test sites in the U.S., France, Britain, Russia and Australia. When deposited in soil, the oxidation process of DU to uranium dioxide (UO_2_) proceeds during the weathering of metal over time. The corrosion progresses produces oxidized uranium species with lower density than the metallic DU the ratio of the molecular volume of the oxide to the molecular volume of metal, the Pilling-Bedworth [6] ratio is greater than 2 for this metal/oxide pair. Because the molecular volume of the oxide is significantly higher than the metal the oxide chips off of the metal surface exposing the underlying metal to the oxidative environment. This non-protective oxide formation continues over time. In some cases, 100 % of the metallic uranium present in soil is converted to the uranium oxide. The less oxidized U (IV) is short lived and further oxidized to U (VI) mineral. Metaschoepite (UO_3_·nH_2_O) is a primary product of DU corrosion [7,8]. These processes of DU corrosion are affected by geochemical and physical properties of the environment, such as soil chemical composition, water saturation conditions, pH, redox potential, nature and concentrations of organic and inorganic materials [8].

Extensive research has been done on the corrosion of uranium metal in different environments, including the atmosphere [9], water [8], soil [5,6,10], hydrogen [11,12], oxygen [13]. Previous research focused on the characterization of DU corrosions in field collected samples from firing test sites and conflict zones [5,14,15]. Handley-Sidhu et al. [8] used microcosms with a range of moisture content to investigate the effect of redox condition and soil water content on the corrosion. However, the method did not facilitate the determination of the corrosion product on the uranium metal and in the soil or their relationship with the surrounding soil. In order to fill this knowledge gap, an experimental technique for controlling experimental variables in order to determine the dependencies of the corrosion rates and processes. The result is the demonstration of a novel method for studying metal corrosion in soil environments.

### Procedures

1 Condition soil at ambient temperature and humidity to desired initial moisture content
2 Grind the soil to pass through 2 mm screen
3 Place weighed soil (W_1_) in a plastic beaker (W_2_) with 0.5 mm-scale holes in the bottom
4 Place beaker in a petri dish of water (maintained at a depth of >2 cm) for capillarity experiment
5 Allow soil to drain until the surface of the soil exhibit a thin layer water film (water transport through the soil by capillary)
6 Weight the beaker with wetted soil (W_3_) and use the following equation to calculate the saturation moisture capacity(SMC) of the soil

SMC = (W_3_ − W_1_ − W_2_)/W_1_ × 100 %

**Fig. 1:**
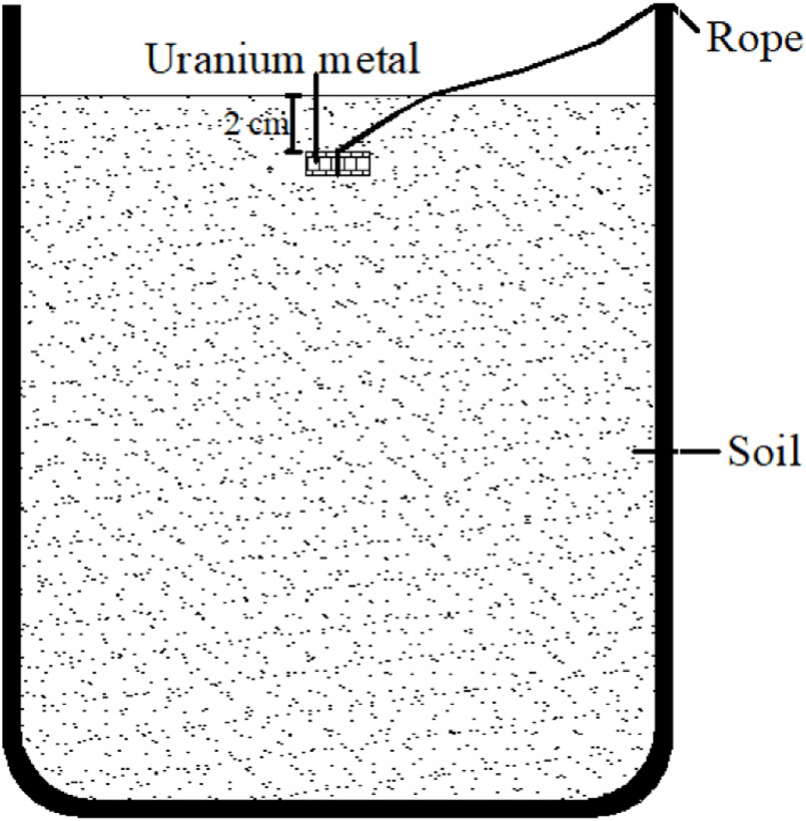
The structure diagram of the corrosion device.
14 Remove the uranium metal on the desired experimental schedule, use brush to remove the attached soil from the surface, and perform XRD, SEM, FT-IR and Raman spectra and other analytical techniques to characterize the uranium metal surface of samples as the weathering process proceeds. Record the mass of the uranium metal at each schedule coupon removal
15 Repeat the step 13 and 14 for as many time intervals as required
16 Dissect each system and collect soil sample at 0−1 cm and 2−3 cm around the uranium metal in horizontal direction
17 Use X-ray fluorescence spectrometer (XRF), XRD and other methods to characterize the coupon and soil
18 Analyze uranium fractionation and total uranium in soils with selective sequential dissolution (SSD) [16]. This SSD method can measure 7 different fractionation uranium of the soil sample. If these data were obtained, it will help us have a better understanding of the transport mechanism of uranium corrosion products in horizontal direction in different regimes.
19 Calculate the corrosion rate (the mass loss was used to calculate the rate, and suppose the surface area of the sample was a constant) of uranium metal in soils with different soil moisture regimes

7 Prepare three clean wide-mouth glass bottles and weigh them
8 Place soil in the bottle with force until the compactness reaches 95 %
9 Weigh the bottle and soil and calculate the mass of soil in each bottle
10 Calculate the amount of water to be added to reach saturation, field capacity moisture (about 60 % saturated moisture) and air dried (or other desired moisture contents)
11 Use X-ray power diffraction (XRD), Scanning Electron Microscopy (SEM), Fourier-transform infrared spectroscopy (FT-IR) and Raman spectra to characterize the uranium metal samples
12 Record the initial mass of uranium metal sample
13 Place uranium metal sample and cover with the desired thickness of overlying soil (Fig. 1)

## Measuring procedures of the total and fractionation of uranium

### Total uranium

1 The total uranium concentration for each sample was measured with Inductively coupled plasma mass spectrometry (ICP-MS).
2 1.0 g soil sample added into 50 mL digestion vessels with 2 replicates
3 25 mL of 4 M HNO_3_ was added in vessel, and the mixture was heated in water-bath at 80℃ for 16 h [17,18]
4 The digest solution is filtered using a 0.45-μm filter
5 Dilute the filtrate in order to produce a sample for analysis within the linear range of the ICP-MS calibration
6 Measure the uranium concentration

### Fractionation

The SSD method [16] was used to test the fraction of uranium in soils

### Final remarks

This work describes a uranium corrosion simulation system for the direct investigation of metal corrosion rates and processes in soil environments under controlled conditions in the laboratory. The procedure includes the preparation of soil with different moisture regimes, assembling the system, placement of uranium metal samples, characterization of uranium metal and soil samples at defined point during the corrosion process. The laboratory technique previous employed [8] was used to establish the level of uranium corrosion at 242 days and 337 days in a soil at field-moisture capacity (Table 1). The method described here provides a means of mass loss determination at a number of times during the corrosion process as well as the changes of sample morphology and mineralogy. The speciation of uranium following corrosion events is possible using the newly developed experimental method. The current method provides a laboratory system simulating the corrosion process of uranium metal in different soil moisture regimes, the use of which allows fuller understanding of the rates and mechanism of uranium metal corrosion and transport processes in soils. This method had the advantages of having low secondary uranium pollution, no energy consumption, ease of operation.

**Table 1:** Time schedule.

Number	Time (week)
1	0
2	1
3	4
4	9
5	16
6	25
7	36
8	49

## Model testing

**Fig. 2:**
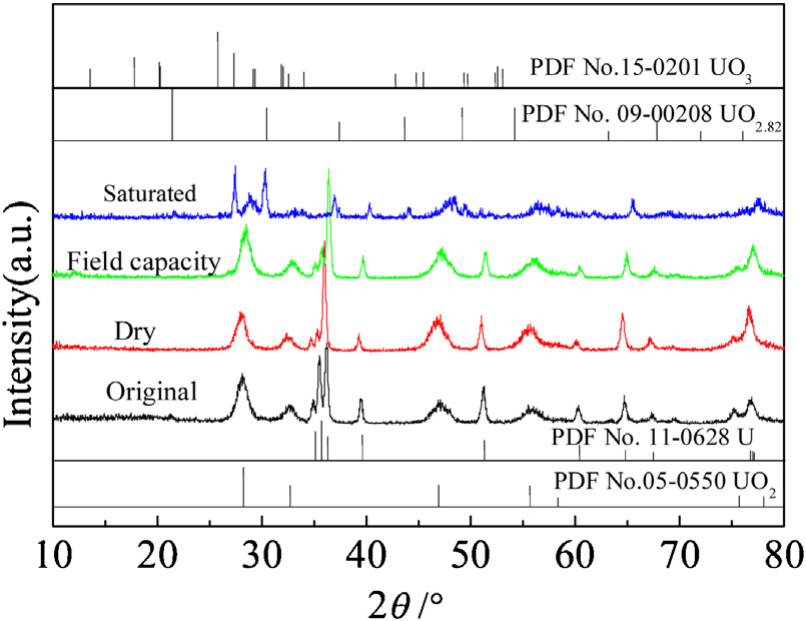
XRD spectra of uranium metal in different soil moisture regimes after 9 weeks.

In order to investigate the effect of moisture regimes on uranium metal corrosion, XRD was used to characterize crystalline phase of uranium metal and its products at certain period (0 and 9 weeks, Fig. 2). The characteristic peaks of U (PDF No.11-0628) and UO_2_ (PDF No. 05-0550) were observed in all samples. In the dry and field capacity moisture regimes, the peak intensity of U located at 35.59° were weak. However, the characteristic peaks of UO_2_ are distinct in the field capacity soil. In the saturated soil, the peaks that developed and located at 21.44°, 30.48°, 43.69° and 27.35° were attributed to the characteristic peaks of UO_2.82_ (PDF No.09-0208) and UO_3_ (PDF No.15-0201), respectively. The corrosion rate was in the sequence of saturated soil > field capacity soil > dry soil. These results confirmed that moisture had a significant effect on the corrosion of uranium metal.

**Fig. 3:**
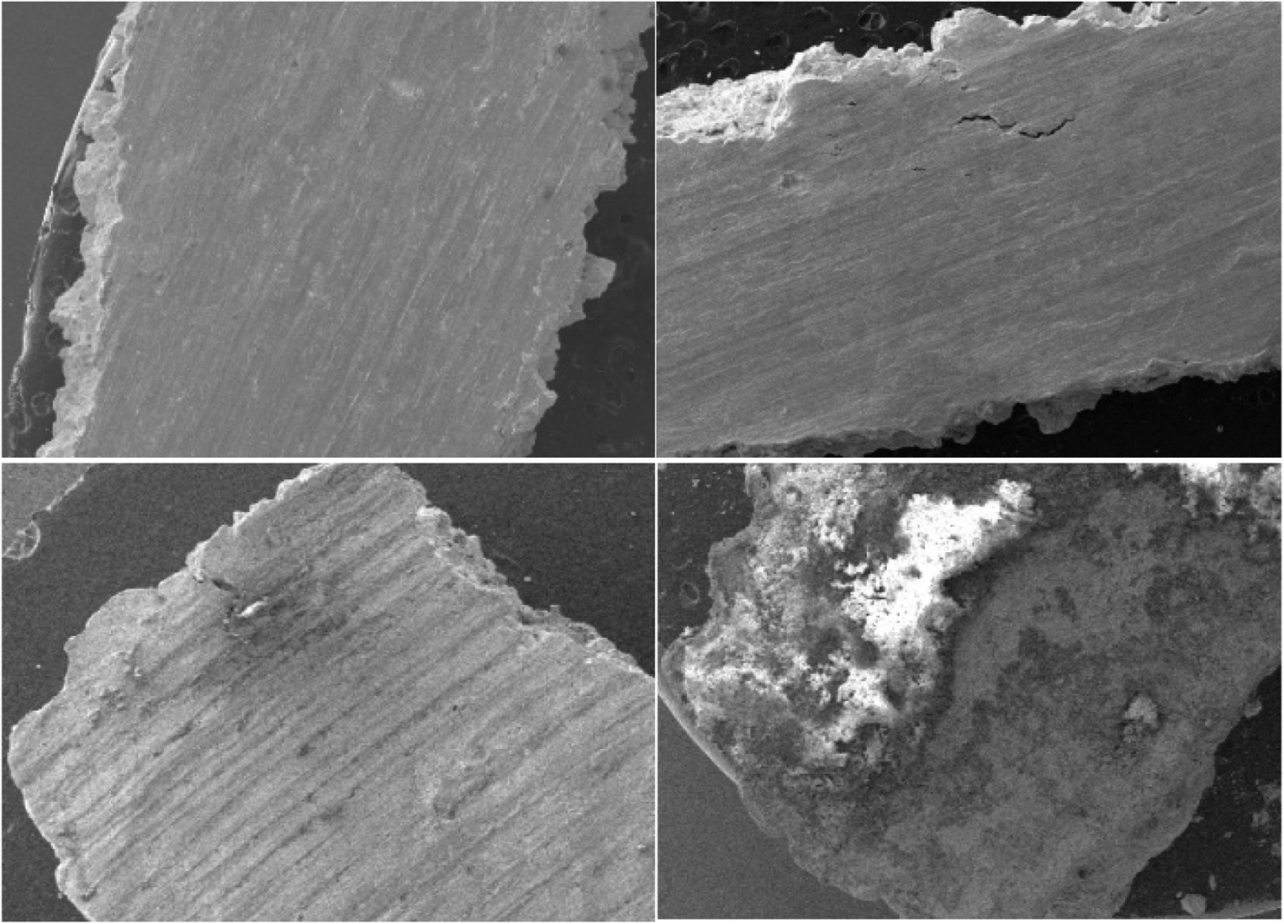
SEM images of uranium metal in different soil moisture regimes after 9 weeks (a-original; b-dry soil; c-field capacity soil; d-saturated soil).

The morphology of uranium metal in different soil moisture regimes were analyzed with SEM (Fig. 3). There was no obvious change of the uranium metal in soils with the dry and field capacity regimes. However, for the saturated soil, the surface and edge of the uranium metal became rough and blunt. Meanwhile, some areas were brighter. This is because some uranium oxides have better conductivity. These results further indicated the fastest corrosion in the saturated soil.

A balance was used to detect mass loss of uranium metal (Table 2). As shown in Table 2, the mass loss of uranium metal in soils with the saturated and field capacity regimes were 52 and 4 times greater than that in the dry soil. This agreed with the results of XRD and SEM.

**Table 2:** The mass loss of uranium metal in different soil moisture regimes after 9 weeks.

Soil types	Mass loss (g)
Dry	0.0011
Field capacity	0.0044
Saturated	0.0572
